# A review of techniques for spatial modeling in geographical, conservation and landscape genetics

**DOI:** 10.1590/S1415-47572009000200001

**Published:** 2009-06-01

**Authors:** José Alexandre Felizola Diniz-Filho, João Carlos Nabout, Mariana Pires de Campos Telles, Thannya Nascimento Soares, Thiago Fernando L. V. B. Rangel

**Affiliations:** 1 Departamento de Biologia Geral, Instituto de Ciências Biológicas, Universidade Federal de Goiás, Goiânia, GOBrazil; 2 Programa de Doutorado em Ciências Ambientais, Universidade Federal de Goiás, Goiânia, GOBrazil; 3 Department of Ecology and Evolutionary Biology, University of Connecticut, Storrs, CTUSA

**Keywords:** autocorrelation, geographical genetics, isolation-by-distance, landscape genetics, spatial regression

## Abstract

Most evolutionary processes occur in a spatial context and several spatial analysis techniques have been employed in an exploratory context. However, the existence of autocorrelation can also perturb significance tests when data is analyzed using standard correlation and regression techniques on modeling genetic data as a function of explanatory variables. In this case, more complex models incorporating the effects of autocorrelation must be used. Here we review those models and compared their relative performances in a simple simulation, in which spatial patterns in allele frequencies were generated by a balance between random variation within populations and spatially-structured gene flow. Notwithstanding the somewhat idiosyncratic behavior of the techniques evaluated, it is clear that spatial autocorrelation affects Type I errors and that standard linear regression does not provide minimum variance estimators. Due to its flexibility, we stress that principal coordinate of neighbor matrices (PCNM) and related eigenvector mapping techniques seem to be the best approaches to spatial regression. In general, we hope that our review of commonly used spatial regression techniques in biology and ecology may aid population geneticists towards providing better explanations for population structures dealing with more complex regression problems throughout geographic space.

## Introduction

Most evolutionary processes occur in a spatial context. The genetic variation originated by random mutations and drifting within local populations will disperse through geographically-mediated gene flow, whereas selection gradients will appear, since environmental factors will also be geographically arranged. Consequently, since the late 1970's, several techniques in spatial analysis started to be used to investigate these processes by analyzing spatial patterns of genetic variation among populations (see Epperson, 2003 and Diniz-Filho *et al.*, 2008a for recent general reviews). In turn, this allowed for the emergence of many slightly different (but highly overlapping) research programs, integrating ecology, evolutionary biology and genetics (Diniz-Filho *et al.*, 2008a). These techniques usually involve the estimation of parameters from spatial structure, such as the geographic distance at which genetic data can be considered independent, which in turn can be linked to ecological or evolutionary processes, such as dispersal. More complex micro-evolutionary inferences can be performed by comparing mapping patterns and their spatial signature, for different alleles and loci (see Sokal and Oden, 1978a,b; Sokal and Wartenberg, 1983; Sokal *et al.*, 1989). Understanding such patterns within species can also be important in optimizing strategies for biodiversity conservation (Diniz-Filho and Telles, 2002, 2006; Diniz-Filho *et al.*, 2006).

Most of these techniques rely on the spatial autocorrelation patterns of genetic variation (Sokal and Oden, 1978a, b). Spatial autocorrelation occurs when closer samples in geographical space tend to be more similar or dissimilar to each other than expected by chance alone, for a given variable such as allele frequencies (Legendre and Legendre, 1998). Spatial autocorrelation in a biological variable can be caused by endogenous processes, in which an intrinsic property of the organisms in spatially distributed populations (such as higher levels of dispersal) causes higher genetic similarity among neighboring locations. Another possibility is that an exogenous factor, in which the genetic variable is responding to variation in an environmental variation, causes the observed pattern (Fortin and Dale, 2005; Kissling and Carl, 2008). In most cases, a combination of these two “types” of factors will influence spatial patterns in biological variables.

In population genetics, autocorrelation has been usually considered as caused by endogenous processes, especially when analyzing neutral markers (although natural selection cannot be ruled out in many instances). Inferences on micro-evolutionary processes have been reached based on parameters extracted from autocorrelation analysis, through a descriptive and exploratory analysis of the spatial structure underlying genetic variation. However, recognition that isolation among populations caused by exogenous effects (including anthropic disturbances) (see Manel *et al.*, 2003; Telles *et al.*, 2007; Storfer *et al.*, 2007; Holderegger and Wagner, 2008; Soares *et al.*, 2008;) can affect neutral loci and create spatial patterns in genetic variation, has led to other widely discussed approaches in spatial analyses in diverse research areas in biology (*i.e.*, ecology and biogeography - see Diniz-Filho *et al.*, 2003, 2007b). The existence of autocorrelation can perturb significance tests and parameter estimates on analyzing data using standard statistical techniques, when a given response variable (genetic data) is modeled as a function of explanatory variables, as for instance, patterns of human occupation or historical effects creating isolation among local populations. In this case, more complex models incorporating the effects of autocorrelation must be used instead of standard and well-known regression and correlation models. The main problem is that spatial autocorrelation in data also causes inferential statistical problems, since Type I errors in regression and correlation analyses are always inflated (see Legendre, 1993). Thus, when dealing with exogenous processes affecting genetic variation, it is important to apply statistical techniques that take into account intrinsic demographic factors and population dynamics creating intrinsic autocorrelation.

Here, we review those modeling techniques which have already been well studied and used in many fields of biology and science in general (see Cressie, 1993; Haining, 1990, 2002; Schabenberg and Gotway, 2005), but only recently have they been mentioned in the contexts population, conservation and landscape genetics (Storfer *et al.*, 2007). We describe these techniques and show their application in a simple simulation of genetic data, in which spatial patterns in allele frequencies were generated by a balance between random variation within populations and spatially-structured gene flow. We show that the avoidance of their use tends to increase Type I errors when relating genetic variation with exogenous factors structured on geographical space.

## Spatial Regression Techniques 

### Spatial autocorrelation in residuals of standard regression models

Suppose that an allele frequency is estimated in local populations and that the purpose/proposal is to model the dependence of this allele frequency on an explanatory variable, such as temperature (when looking for selection gradients) or intensity of anthropogenic effects that, for example, could create patterns through increasing isolation. The standard approach to analyze this kind of data is to perform a linear regression of allele frequencies (Y) against the explanatory variable (X), so that the observed frequency in each *i*th population can be expressed by:


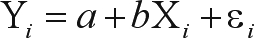
(1)

where *a* and *b* are the linear (intercept) and angular (slope) coefficients and ε_*i*_ is the residual term, given by the difference between observed and expected frequency of the population *i*. In a matrix form, the equation above can be written (and generalized) by:


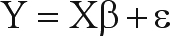
(2)

where β is the vector with coefficients associated with *k* explanatory variables (plus the intercept term β_0_ or *a*). Thus, the R^2^ of this regression model, given by the ratio between predicted and observed sum of squares, will provide the amount of variation in allele frequency that is “explained” by the explanatory variables. It is assumed that the ε term is normally distributed with constant variance, and is independently distributed among observations, so that covariance matrix **C** among residuals is equal to:


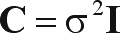
(3)

where σ^2^ is the variance of the residuals, which is constant throughout the diagonal of **C**, and **I** is an identity matrix. Under these assumptions, the coefficients in the vector β can be obtained by:


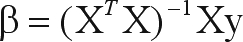
(4)

These coefficients are usually estimated by using least-square techniques, and this simple non-spatial regression model will be called here Ordinary, or non-spatial, Least-Squares (OLS) (which is actually the general method of estimating β). However, a higher dispersal or migration will link populations closer in geographical space, so that any single stochastic variation will be shared among adjacent populations, and their similarity will be explained by these stochastic processes and not by their common response to X. Thus, close populations in geographic space (*i.e.*, which are linked by higher levels of gene flow) show similar deviations from expected allele frequency by effects of X. This problem can be formally evaluated by checking whether the residuals E of the model for local populations closer in geographic space are more similar than expected by chance alone. In other words, this can be evaluated by estimating spatial autocorrelation in model residuals ε.

Although autocorrelation at short distances will not generate broad scale gradients, except if coupled with some form of historical effects, autocorrelation among residuals will actually generate an overestimation of residual degrees of freedom, thus completely disturbing any significance tests associated with the model. Even under alternative frameworks for model evaluation, such as the information theory (see Burnham and Anderson, 2002), model choice will be perturbed by residual autocorrelation (Diniz-Filho *et al.*, 2008b).

The residual autocorrelation can be evaluated using several techniques (see Sokal and Oden, 1978a,b, Legendre and Legendre, 1998), but the most commonly applied approach in population genetics is to estimate Moran's *I* autocorrelation coefficients, given as:


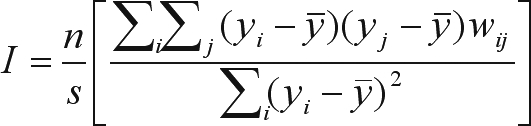
(5)

where *n* is the number of samples (local populations), *y*_*i*_ and *y*_*j*_ are the values of the allele frequencies (or any quantitative trait) measured in the populations *i* and *j*, *y* is the average of *y* and *w*_*ij*_ is an element of the **W** matrix. In this **W** matrix, the elements are equal to 1 if the pair *i*, *j* of local populations is within a given distance class interval (indicating populations that are “connected” in this class), and otherwise *w*_*ij*_ = 0. *S* indicates the number of entries (connections) in the **W** matrix. The value expected under the null hypothesis of the absence of spatial autocorrelation is -1/(*n* - 1).

In practice, Moran *I* is usually calculated by using different distance classes, connecting, in the **W** matrix, pairs of local populations situated at increasing geographic distances. Thereby, a sequence of coefficients is obtained and a spatial correlogram appears when they are plotted against geographic distance classes. This correlogram better describes the complexity of spatial patterns, both in original variable and model residuals. Most evolutionary inferences using autocorrelation in population genetics and phylogenetic comparative analyses have been performed based on correlograms, although these were not obtained from model residuals, but instead from original allele frequencies or phenotypes (Sokal and Oden, 1978a, b; Sokal and Wartenberg, 1983; Sokal *et al.*, 1989; Diniz-Filho and Malaspina, 1995; Diniz-Filho, 2001, 2004).

The statistical significance of Moran's *I* can be obtained by estimating its variance, under different assumptions and obtaining a standard normal deviation statistics Z. For model residuals, these formulae do not apply exactly (see Schabenberg and Gotway, 2005), and so significance levels can be established by randomization techniques (Manly, 1997). Another recent development is to apply local versions of Moran's *I*, in which a spatial autocorrelation coefficient is calculated for each spatial unit, thereby revealing how similar neighbouring values are regarding each of these “focal” spatial units (Sokal *et al.*, 1998a, 1998b; Fotheringham *et al.*, 2002). This is a more forceful way of evaluating more localized spatial patterns in model residuals, thus allowing for a better understanding of genetic variation and greater ability in detecting problems in regression models.

Mantel tests (Mantel, 1967; Manly, 1985, 1997) have also been widely used in population genetics for comparing geographic and genetic distances. In this context of spatial regression, multiple Mantel tests (Smouse *et al.*, 1986; Manly, 1985) could be used to evaluate the effects of different sets of explanatory matrices X in pairwise genetic distances. However, this is basically a partial regression model (see below) in a matrix design, and has mainly been used for exploring relationships and not correcting statistical inference.

Once autocorrelation in model residuals is detected, a number of modifications in Eq. (1) can be performed taking this into account, both in order to improve understanding of genetic variation, as well as to better estimate and test model parameters. In general, we will refer to these subsequent models, as reviewed below, as “spatial regression models”. These can be grouped into two classes, based on the idea of incorporating autocorrelation either into a model structure or into model residuals. Since the problem of modeling spatially-structured genetic data appears when autocorrelation exists in model residuals, as described above, the solution to the problem is exactly to eliminate this autocorrelation. This can be statistically achieved by two different approaches (see Martins and Hansen, 1996): 1) it is possible to introduce into the model structure certain spatial “terms”, such as additional vectors in X which are other variables that capture spatial variation, so that E becomes independently distributed; or 2) assume that ε is autocorrelated, and explicitly incorporate this when estimating coefficients in β. Both classes of models will be discussed in more detail below.

### Incorporating geographic space in model structure

There are many ways of incorporating spatial variables into the model structure to eliminate residual autocorrelation. This can be expressed by a general model of the form:


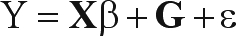
(6)

where **X**, β and ε are as defined for Eq. (2) and **G** is a vector or matrix (*i.e.*, spatial terms and associated spatial coefficients) expressing geographic space or, more appropriately, the geographically-structured genetic variation among local populations. Thus, this class of spatial regression tries to “filter” or eliminate autocorrelation in model residuals by capturing it in the **G** term of Eq. (6). Therefore, the problem is how to define “space” in Eq. (6) and express it in **G** terms.

The first and simplest way of defining space is by directly using the spatial coordinates of populations (*i.e.*, latitude and longitude) that can be added as spatial predictors, so that:


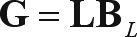
(7)

where **L** is a vector with spatial coordinates of local populations and **B**_*L*_ are the slopes of these coordinates. What Eq. (7) is actually doing is to express part of genetic variation, such as a north-south cline, as a plane in geographic space. The spatial component in Eq. (7) can be changed by adding polynomial expansions, thereby adjusting to quadratic or cubic functions of spatial coordinates. This technique is known as “trend surface analysis” (see Legendre and Legendre, 1998), and is better designed to model broad scale trends and not local autocorrelation in model residuals. Anyhow, these can be useful if genetic variation is in part caused by broad-scale effects, such as directional selection gradients caused by environmental factors (such as temperature) and is structured at these scales, or by colonization historical events with strong directional components (see Bocquet-Appel and Sokal, 1989).

Another way to express more localized spatial patterns is by an autoregressive term. There are several forms to express autoregressive models, but the main idea is that the response variable Y can be modeled as:


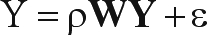
(8)

where ρ is an autoregressive coefficient and **W** is a matrix expressing spatial weightings, or rather, how one local population affects the other. The elements of **W** can be defined in many ways, including connectivity (as in **W** matrices of a spatial correlogram using Moran's *I*) or by the inverse of geographic distances *d*_*ij*_ among local populations (*w*_*ij*_ = 1/*d*_*ij*_). It is also possible to use another term to increase the complexity of the relationship between weights and distances, so that *w*_*ij*_*= 1/d*_*ij*_, where α is a coefficient that controls curvilinearity in the relationship between geographic distances and weights. Thus, the above term ρ**WY** is the estimated value of Y in a given local population if its genetic variation is a function of nearby local populations weighted by their geographic distances (expressed as weights). Thus, the term **G** in the above equation can be expressed as the vector ρ**WY**, so that:


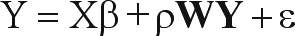
(9)

This model is usually called the “lagged-response autoregressive model”. Alternatively, it is possible to incorporate autoregressive terms, as defined above, for both Y and X variables, so that the overall Eq. (6) becomes:


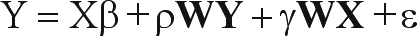
(10)

where γ are the spatial autoregressive coefficients ρ for each explanatory variable. This model is usually called the “lagged-predictor or mixed autoregressive model”.

A different approach to incorporating space into models is to extract eigenvectors from a matrix expressing the spatial relationship among local populations, and to use part to establish the term **G** of Eq. (6). This approach have been called eigenvector-based spatial filtering, the principal coordinate of neighbor matrices (PCNM), or, and in general, spatial eigenvector mapping (SEVM) (see Borcard and Legendre, 2002; Borcard *et al.* 2004; Griffith, 2003; Diniz-Filho and Bini, 2005; Griffith and Peres-Neto, 2006). The basic difference among these slightly different applications is from which matrix expressing geographic space, the eigenvectors are to be extracted. Diniz-Filho *et al.* (1998) also proposed to extract eigenvectors from phylogenetic distance matrices, calling this process phylogenetic eigenvector regression (PVR), and using this to express phylogenetic components in a trait Y measured across species (or populations, as seen in Diniz-Filho *et al.*, 1999; see also Diniz-Filho *et al.*, 2007a for a more complex combination of spatial and phylogenetic mapping).

Eigenvectors of a spatial matrix express the relationships among local populations at decreasing spatial scales, so that first eigenvectors (*i.e.*, those associated with large eigenvalues) tend to express broad-scale structures, whereas eigenvectors with small eigenvalues tend to express local patterns. Thus, the advantage of eigenvector mapping is the flexibility in dealing with patterns at multiple scales, and the possibility of iteractively improving modeling process by adding or removing these eigenvectors. However, this may also pose a problem, since a very large number of eigenvectors (*i.e.*, *n* - 1) exists, so there must be a certain criterion for establishing which are to be used in the model. This is the same as the “stopping-rule problem” in multivariate analysis for deciding which eigenvectors are meaningful (see Legendre and Legendre, 1998). Several criteria can be used, but in this modeling context the most important is to parsimoniously select the smallest number of eigenvectors that ensure a minimum desirable level of spatial autocorrelation in residuals.

### Incorporating autocorrelation in model residuals

The second class of spatial regression does not attempt to minimize residual autocorrelation by “filtering” it from variable Y, as described above. Instead, the idea is to solve the problem by incorporating spatial autocorrelation as part of residual variation, and correcting (or generalizing) the way coefficients in β and their variances are to be estimated. The basic idea is actually based on Eq. (4) above. Actually, Eq. (4) is a simplification of a more general equation of the form:


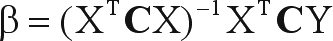
(11)

where **C** is a covariance matrix expressing the relationships between local populations, quite similar (or analogous) to **W**. Notice that, if there is no autocorrelation in residuals, and variances are homocedastic, the **C** matrix becomes a single number (σ^2^), so that Eq. (10) is reduced to Eq. (4).

Once again, the different techniques that can be found in the literature are named after different ways of defining **C**. Wagner *et al.* (2005) also used a similar approach to generalize the AMOVA, a widely used technique in population genetics. The most widely used techniques are simultaneous (SAR) and conditional (CAR) spatial autoregressive models, based on p autoregressive coefficients and the **W** matrix (see Wall 2004), and similar to those defined above, in which the **C** matrix is given by:


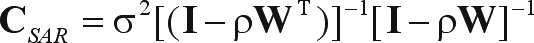
(12)

and


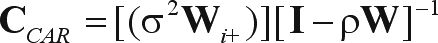
(13)

Another related model, called the moving average (MA), can be obtained by defining C as:


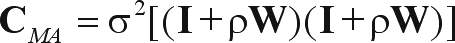
(14)

Equations (8) to (10) are also forms of simultaneous autoregressive models, but since they are based on the “filter” approach, they are called lagged-models, whereas the simultaneous form presented in Eq. (11) is sometimes referred to the SAR error model (Kühn, 2007, Dormann *et al.*, 2007, Kissling and Carl, 2008).

Finally, it is very important to note that success in the application of these techniques is not always guaranteed, because of model-fit problems. For example, if the spatial structure way, as expressed in the **W** matrix, does not capture those spatial processes underlying genetic variation, then the residual can still possess spatial autocorrelation. Thus, it is important to use Moran's *I* or some other autocorrelation coefficient to test whether the assumption of spatial independence of residuals is being violated or not.

## A Simple Simulation 

We showed the relative performance of the models described above, by using a simple simulation of an isolation-by-distance process in geographic space, generated with EASYPOP 2.0 (Balloux, 2001). The simulation consisted of a total of 30 local populations, each with 20 diploid individuals (10 males and 10 females), with a known spatial distribution (see below). Dispersal distance was equal to 2 units, and a maximum of 10 alleles per locus was generated under an infinite allele model, with maximum variability. Gene dynamics occurred throughout 500 generations. Thus, spatial patterns that appear in genetic data (allele frequencies) were generated by a purely spatially-structured stochastic process combining mutation, drift and gene flow, without exogenous effects.

**Figure 1:**
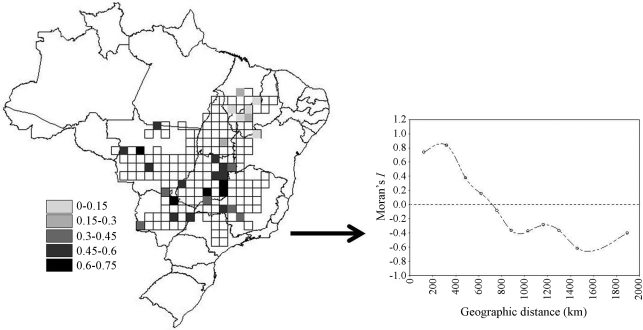
Allele frequency of each of the 30 populations in the Cerrado biome, and a spatial correlogram, placing in evidence the high positive autocorrelation in the first distance classes.

As a reference for geographical dimension, the 30 populations were randomly assigned to a grid with 181 cells covering the Cerrado biome (Figure 1). The Cerrado is the second largest biome in Brazil (the first is the Amazon Rain Forest), occupying more than 1,500,000 km^2^, comprising a mosaic of different vegetation types dominated by a tropical savanna matrix, but also ranging from open grasslands and rocky fields to dense woodlands and dry forests (Oliveira-Filho and Marquis, 2002). The allele frequencies were then modeled by using spatial regression techniques as a function of the main directions of spatial variation in human occupation throughout the biome, these being derived from a factor analysis of 23 socio-economic variables, surrogates of modernization in farming, cattle breeding and human demography (see Rangel *et al.*, 2007 for details). Take note that we are not simulating any effect of these factors on allele frequencies, and spatial patterns in genetic data are only generated by endogenous processes. Thus, statistically significant regression coefficients express the pure coincidence of spatial patterns in data (a north-south directed cline in human demography) or an inflated Type I error of the different models under spatial autocorrelation in the data. All spatial analyses were performed using the SAM 3.0 (Spatial Analysis in Macroecology; Rangel *et al.* 2006) program.

As foreseen, allele frequencies showed a significant spatial pattern, with an expected spatial correlogram under isolation-by-distance, with high positive autocorrelation coupled with negative or stabilizing autocorrelation in the last-distance classes (Figure 1). When modeling the allele frequencies as a function of the three factors of human occupation (explanatory variables X), one would expect no significant relationships to arise. However, on using the standard OLS regression, out of the 20 models obtained, 14 contained at least one significant coefficient, and out of 60 regression slopes, a total of 29 were significant at the 5% probability level (Table 1). By chance alone, one would expect to find 1 out of 20 models with some significant coefficients, or rather 3 coefficients out of the 60 tested. Thus, despite the absence of causal relationships between Y and X, the OLS tend to disclose many significant relationships between genetic variation and exogenous processes.

Repeating these analyses, using the 7 different spatial regression models, gave mixed results, when counting the number of significant models and coefficients. For autoregressive models, elements in the **W** matrix were defined as *w*_*ij*_*= 1/d*_*ij*_^3^ (where *d*_*ij*_ are the distances between cells), and for PCNM the eigenvectors used in the model were those with significant spatial patterns (Moran *I* in the first distance class > 0.1), with truncation distance equal to 250 km. In general, spatial regression models performed better than OLS, both in terms of frequency of models and frequency of coefficients, the two best models being LagRES and PCNM, with a frequency of significant coefficients equal to 13% and 18%, respectively. However, some spatial regression methods, such as CAR, performed even worse than OLS.

A “distance” from null expectation can also be obtained for each method, by the sum of squares of standardized slopes or each explanatory variable, assuming that expected slopes are zero. According to this metric, SAR, MA, LagRES and PCNM gave lower distances than OLS, thus being less affected by autocorrelation and furnishing results closer to the expected under the null hypothesis (a null vector of slopes).

**Figure 2:**
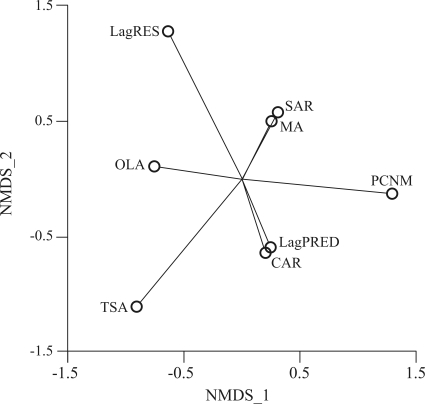
Distribution of spatial regression methods in the 2D solution of non-metric multidimensional scaling (NMDS) based on their standardized slopes. The methods were: Ordinary Least-Squares (OLS); Principal Coordinate of Neighbor Matrices (PCNM); Lagged Response (LagRES); Lagged Predictor (LagPRED); Simultaneous Autoregression (SAR); Conditional Autoregression (CAR); Moving Average (MA); Trend Surface Analysis (TSA).

Finally, an ordination using a non-metric multidimensional scaling of distances among methods, and based on their standardized slopes, supports the above patterns. LagRES, PCNM and TSA are the most diverse methods, at extreme positions in ordination space (Figure 2), whereas TSA is somewhat closer to OLS. The other methods are at intermediate positions.

## Discussion

Our analyses agree with the recent comparative evaluation by Bini *et al.* (2009), in the sense that the performance of spatial regression models is quite idiosyncratic and data-dependent, at least in terms of parameter estimates. From our analyses, it is evident that spatial filtering approaches (especially LagRES and PCNM) seem to work better for our simulated data than those incorporating autocorrelation in model residuals, a result opposed to a slight trend found by Bini *et al.* (2009) on analyzing 99 macro-ecological datasets (although they also found a better performance by SEVM, analogous to PCNM). This may be due to the strong endogeneous component in our simulated data, whereas in Bini *et al.* (2009), macro-ecological data exogenous components are usually dominant (see also Hawkins *et al.*, 2007). The same is valid for simulations performed by Dormann *et al.* (2007) and Kissling and Carl (2008). In all these ecological analyses, LagRES was the worst model, whereas here it was the model with the lowest type I errors. At the same time, our performed simulations are constrained by the shape of the Cerrado domain, so that common clinal patterns may appear alone and by chance, even without a causal basis, and it is difficult to tease these effects apart.

Because of the relatively great number of significantly large coefficients and models found in our analyses (a minimum of 13% for LagRES), one could argue that spatial regression models, although tending to perform better than OLS, are not entirely effective in decoupling the endogenous and exogenous processes driving allele frequencies. This is true, although it is not necessarily due to statistical problems with methods, but instead to conceptual problems underlying correlation and causation (Shipley, 2000). It is important to note that, in our simulations, statistically significant coefficients or models purely express the coincidence of spatial patterns in data, or an inflated Type I error of the different models, because of residual spatial autocorrelation. We simulated stochastic patterns in allele frequencies and used real patterns of human occupation in the Cerrado as explanatory variables, thereby following recent approaches in ecological data (Dormann *et al.*, 2007; Dormann, 2007; Kissling and Carl, 2008). Although this approach is more realistic, it also opens the possibility of common trends appearing by chance alone, since independent spatial patterns are not simulated in both Y and X variables. For example, if allele frequencies under isolation-by-distance tend to form a cline, the spatial configuration of the Cerrado alone, itself more oriented across a north-south axis, would be enough to generate a correlation with the north-south cline in human demography, even if these two patterns are not intrinsically related. Spatial regressions are mainly designed to deal with inflated Type I errors due to/because of short-distance autocorrelation, and would not solve broad-scale associations, so it would be conceptually impossible to distinguish between causal effects when similar trends appear in data, even if they are originated by different mechanisms. This is a general problem of all observation (not experimental) data (see Shipley, 2000), and is not a problem of particular modeling approaches.

Thus, part of the much higher Type I errors that appeared in our analyses were due to a north-south cline that arose in both allele frequencies (because of the spatial configuration of local populations in the simulations) and human demography. Indeed, if this last explanatory variable is not included in the analyses and the frequency of significant models and coefficients are recalculated (Table 2), it is possible to see that models are closer to null expectation (*i.e.*, zero slopes for the predictors). Also, Type I error of OLS increases to 40%, whereas Type I errors of spatial regression models are reduced to much more acceptable levels, equal to 7.5% for LagRES (see Diniz-Filho and Torres, 2002 and Martins *et al.*, 2002 for analogous Type I errors estimated in comparative analyses) (Table 2). Notice that when an improved performance appears, it mainly occurs with “filtering” methods that remove the common trends, and not with methods that deal with short-distance autocorrelation in model residuals.

Despite the somewhat idiosyncratic results of comparing spatial regression models in the literature (Bini *et al.*, 2009), there is a consensus that spatial autocorrelation affects and perturbs Type I errors and that, in this situation, OLS does not provide minimum variance estimators (as shown here in our simple simulations). In the recent developments in landscape and conservation genetics, genetic data is usually regressed against sets of explanatory variables to detect factors associated with population structure. So a warning against these undesirable effects in spatial autocorrelation is necessary. Among the techniques tested, PCNM and LagRES performed better with our simulated data, although recently, LagRES has been the subject of criticism in several papers (Dormann *et al.*, 2007; Kissling and Carl, 2008). Due to its flexibility and capacity to deal simultaneously with problems in Type I error and parameter estimation, we reinforce the notion that PCNM and related eigenvector filtering techniques seem to constitute the best approach for spatial regression. In general, we hope that our review of certain spatial regression techniques that have been more commonly applied in biology and ecology to solve autocorrelation “problems”, may help population geneticists to provide better explanations for population structure dealing with more complex regression problems throughout geographic space.

## Figures and Tables

**Table 1:** A comparison of spatial regression methods based on the analysis of null expectation, by regressing allele frequencies evolving under a pure isolation-by-distance process against three explanatory variables (factors). N. models refers to the frequency (out of 20 simulations) with at least one significant (p < 0.05) regression slope, whereas N. coeff. shows the frequency (out of 60 coefficients) of significant coefficients. The Dist(H0) refers to the average Euclidian distances between the regression coefficient vector β and the null expectation (all slopes are zero).

	N. models	N. coeffs	Dist (H0)
OLS	0.65	0.38	0.140
TSA	0.75	0.33	0.170
PCNM	0.50	0.18	0.154
LagRES	0.40	0.13	0.076
LagPRED	0.40	0.20	0.113
SAR	0.70	0.35	0.118
CAR	0.70	0.38	0.138
MA	0.70	0.35	0.116

**Table 2:** The same analyses shown in Table 1, but regressing allele frequencies evolving under a pure isolation-by-distance process against two out of three explanatory variables (removing human occupation).

	N. models	N. coeffs	Dist (H0)
OLS	0.55	0.40	0.069
TSA	0.35	0.25	0.073
PCNM	0.10	0.07	0.103
LagRES	0.15	0.07	0.033
LagPRED	0.20	0.12	0.055
SAR	0.40	0.30	0.060
CAR	0.50	0.45	0.070
MA	0.40	0.30	0.058
